# Coumarin Ameliorates Impaired Bone Turnover by Inhibiting the Formation of Advanced Glycation End Products in Diabetic Osteoblasts and Osteoclasts

**DOI:** 10.3390/biom10071052

**Published:** 2020-07-15

**Authors:** Eun-Jung Lee, Min-Kyung Kang, Yun-Ho Kim, Dong Yeon Kim, Hyeongjoo Oh, Soo-Il Kim, Su Yeon Oh, Woojin Na, Young-Hee Kang

**Affiliations:** Department of Food Science and Nutrition and The Korean Institute of Nutrition, Hallym University, Chuncheon, Kangwon-do 24252, Korea; reydmswjd@naver.com (E.-J.L.); mitholy@hallym.ac.kr (M.-K.K.); royalskim@hallym.ac.kr (Y.-H.K.); ehddus3290@naver.com (D.Y.K.); ohhyeongju@gmail.com (H.O.); ky4850@naver.com (S.-I.K.); suy0411@naver.com (S.Y.O.); nsm0729@hanmail.net (W.N.)

**Keywords:** advanced glycation end-product, bone remodeling, bone turnover, coumarin, glucose, osteoblasts, osteoclasts

## Abstract

Accumulating evidence demonstrates that the risk of osteoporotic fractures increases in patients with diabetes mellitus. Thus, diabetes-induced bone fragility has recently been recognized as a diabetic complication. As the fracture risk is independent of the reduction in bone mineral density, deterioration in bone quality may be the main cause of bone fragility. Coumarin exists naturally in many plants as phenylpropanoids and is present in tonka beans in significantly high concentrations. This study investigated whether coumarin ameliorated the impaired bone turnover and remodeling under diabetic condition. The in vitro study employed murine macrophage Raw 264.7 cells differentiated to multinucleated osteoclasts with receptor activator of nuclear factor-κΒ ligand (RANKL) in the presence of 33 mM glucose and 1–20 μM coumarin for five days. In addition, osteoblastic MC3T3-E1 cells were exposed to 33 mM glucose for up to 21 days in the presence of 1–20 μM coumarin. High glucose diminished tartrate-resistant acid phosphatase activity and bone resorption in RANKL-differentiated osteoclasts, accompanying a reduction of cathepsin K induction and actin ring formation. In contrast, coumarin reversed the defective osteoclastogenesis in diabetic osteoclasts. Furthermore, high glucose diminished alkaline phosphatase activity and collagen type 1 induction of osteoblasts, which was strongly enhanced by submicromolar levels of coumarin to diabetic cells. Furthermore, coumarin restored the induction of RANK and osteoprotegerin in osteoclasts and osteoblasts under glucotoxic condition, indicating a tight coupling of osteoclastogenesis and osteoblastogenesis. Coumarin ameliorated the impaired bone turnover and remodeling in diabetic osteoblasts and osteoclasts by suppressing the interaction between advanced glycation end product (AGE) and its receptor (RAGE). Therefore, coumarin may restore optimal bone turnover of osteoclasts and osteoblasts by disrupting the hyperglycemia-mediated AGE–RAGE interaction.

## 1. Introduction

The skeleton is a metabolically active organ that undergoes continuous remodeling throughout life [1]. Bone regeneration persists even after skeletal maturity as the old bone is replaced by newly formed bone at the same location [2]. Bone remodeling entails depletion of mineralized bone by osteoclasts, followed by the formation of bone matrix by osteoblasts that are subsequently mineralized [3,4]. The remodeling cycle comprises five well-known sequential phases: activation, osteoclast-mediated old bone resorption, reversal, osteoblast-mediated new bone formation, and termination [5]. Bone remodeling supports bone architecture to meet mechanical needs, and interferes with the accumulation of old bone by repairing damaged bone matrix [6]. Bone remodeling is regulated by the parathyroid hormone, calcitriol, glucocorticoids, bone morphogenetic proteins, cytokines, and growth factors [3]. The processes of bone resorption and formation are tightly coupled via the receptor activator of NF-kappa B (RANK)/receptor activator of the NF-kappa B ligand (RANKL)/osteoprotegerin (OPG) system, resulting in a wave of bone formation following each cycle of bone resorption [3,7]. The physiological process of bone remodeling can be derailed by various factors including menopause-associated hormonal depletion, age-related factors, altered physical activity, drugs, and secondary diseases [8]. Impaired bone remodeling frequently results in the failure of systemic mineral homeostasis and progression to bone diseases such as osteoporosis [8,9].

In several metabolic diseases, bone pathology is associated with impaired bone development or bone remodeling [8,10]. Diabetic complications and osteoporotic fracture are chronic diseases that share several features including genetic susceptibility and environmental factors [11]. In addition, both glycemic and bone homeostasis are known to be under the influence of common regulatory factors such as insulin, peroxisome proliferator-activated receptor-gamma, gastrointestinal hormones, and osteocalcin [11,12,13]. Advanced glycation end products (AGE) accumulate in diabetic dysfunction and bone homeostasis [12]. Accordingly, the association between diabetes mellitus and bone health has long been a matter of debate. However, several studies have shown that diabetic bone is characterized by abnormal alterations in bone metabolism, decreased bone mineral content and density, increased fracture rate, and delayed fracture healing [13,14,15]. Furthermore, in diabetes mellitus, bone turnover is reduced or disrupted in parallel with increased risk of bone fractures, in which bone formation declines with reduced mineralization and bone cell number [12,16]. In general, patients with diabetes carry decreased levels of biochemical markers of bone turnover [13,17]. Formative biochemical markers of bone turnover including osteocalcin and alkaline phosphatase (ALP) are reduced, and resorptive biochemical markers such as *C*-terminal markers of cross-links of collagen also decrease in patients with diabetes [13,17,18]. Subsequently, diabetes leads to increased rate of bone loss, altered bone structure, and predisposition to bone fragility due to reduced bone turnover [12].

The increase in fracture risk of diabetic patients depends on older age, duration of diabetes reduced muscle mass, diabetic complications, poor glycemic control, hypoglycemia, and treatment with anti-diabetic medications such as thiazolidinediones, sodium-glucose transport protein 2 inhibitors, and insulin [19,20]. Anti-osteoporotic medications appear to be equally effective in individuals with and without diabetes despite the reduced bone turnover in patients with diabetes [21]. Thiazolidinediones used in anti-diabetic treatment induce preferential differentiation of mesenchymal stem cells into adipocytes rather than osteoblasts, leading to decreased bone formation and increased adipogenesis [22]. Optimal glycemic control and prevention of diabetic complications are therapeutic mainstays to lower fracture risk, although thiazolidinediones enhance fracture risk in postmenopausal women diagnosed with type 2 diabetes [23]. Insulin has an anabolic effect on bone [23]. However, the fracture risk is elevated in type 2 diabetes despite hyperinsulinemia [23]. Furthermore, the weakening of bone tissues may be associated with collagen glycation and AGE accumulation, leading to impaired bone cell function and extracellular matrix (ECM) complications [12]. Diabetes affects bone metabolism and therefore, individual pharmacological targets in anti-diabetic therapies affect the bone quality via indirect effects on bone cell differentiation and bone remodeling. This study investigated whether hyperglycemia-mediated AGE accumulation is a risk factor for osteopathy and osteoporosis due to impaired bone remodeling.

Based on studies demonstrating that specific coumarin derivatives act as potential anti-diabetic agents [24], this study investigated the osteoprotective role of coumarin in diabetes-associated bone diseases. In fact, coumarin displays beneficial anti-osteoporotic activity [25,26]. Little is known about the effects of coumarin on impaired bone remodeling in diabetes. The current study examined whether the impaired bone turnover in diabetes was mediated via hyperglycemia-induced interaction between AGE and RAGE. Additionally, this study explored whether coumarin had a potential role in boosting bone turnover and remodeling of osteoclasts and osteoblasts differentiated from MC3T3-E1 cells upon exposure to glucose.

## 2. Materials and Methods

### 2.1. Chemicals

Fetal bovine serum (FBS), trypsin–ethylenediaminetetraacetic acid, and penicillin–streptomycin were obtained from BioWhittaker (San Diego, CA, USA). 3-(4,5-Dimetylthiazol-yl)-diphenyl tetrazolium bromide (MTT) was purchased from DUCHEFA Biochemie (Haarlem, The Netherlands). Minimum essential medium alpha medium (α-MEM), Dulbecco’s Modified Eagle Medium (DMEM), d-glucose, and Alizarin red S dye were supplied by Sigma-Aldrich Chemical (St. Louis, MO, USA) as were all other reagents unless specifically stated otherwise. Recombinant murine sRANKL was purchased from PeproTech (Rocky Hill, NJ, USA). The carbonic anhydrase II (CAII, cat. no. MAB2184) antibody was provided by R&D System (Minneapolis, MN, USA). Advanced glycation end product-bovine serum albumin (AGE-BSA) protein was obtained from Merck Millipore (Burlington, MA, USA). AGE antibody (cat. no. bs-1158R) was provided by Bioss Antibodies (Woburn, MA, USA). Antibodies of vacuolar H^+^-ATPase (V-ATPase, cat. no. sc-517031), receptor for AGE (RAGE, cat. no. sc-365154), receptor activator of nuclear factor κ B (RANK, cat. no. sc-374360), cathepsin K (cat. no. sc-48353), and collagen type I (cat. no. sc-293182) were obtained from Santa Cruz Biotechnology (Dallas, TX, USA). Antibodies of OPG (cat. no. ab183910) and chloride channel 7 (ClC7, cat. no. ab86196) were supplied by Abcam (Cambridge, UK). Horseradish peroxidase (HRP)-conjugated goat anti-rabbit IgG, goat anti-mouse, and donkey anti-goat IgG were obtained from Jackson ImmunoResearch Laboratories (West Grove, PA, USA).

Coumarin (Sigma-Aldrich Chemical) was dissolved in dimethyl sulfoxide (DMSO) for live culture with cells; a final culture concentration of DMSO was <0.5%.

### 2.2. Culture of Raw 264.7 Cells and Osteoclast Differentiation

Murine macrophage Raw 264.7 cell line (ATCC TIB-71) was obtained from the American Type Culture Collection (Manassas, VA, USA) and routinely cultured in DMEM supplemented with 10% FBS, 100 U/mL penicillin, and 100 μg/mL streptomycin at 37 °C with 5% CO_2_ in air. To differentiate macrophages into mature osteoclasts, Raw 264.7 cells were seeded on a 24-well plate at the density of 1 × 10^4^ cells/mL and incubated in α-MEM (containing 5.5 mM or 33 mM glucose) with 50 ng/mL RANKL in the absence or presence of 1–20 μM coumarin for five days. Culture media were freshly changed every two days, and mature osteoclasts were observed on day 5 [27,28]. 

**Figure 1:**
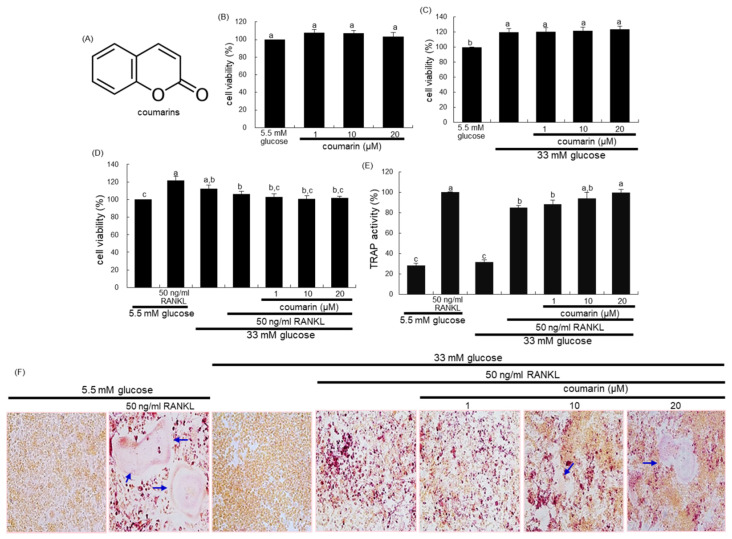
Chemical structure of coumarin (**A**), cell cytotoxicity of coumarin in osteoclastic Raw 264.7 cells (**B**–**D**), tartrate-resistant acid phosphatase (TRAP) activity (**E**), and TRAP staining (**F**). Raw 264.7 cells were cultured in an α-MEM media containing 5.5 mM glucose or 33 mM glucose for two days in the absence and presence of 1–20 μM coumarin or 50 ng/mL receptor activator of the NF-kappa B ligand (RANKL). Cell viability was measured by the MTT assay and expressed as percent cell survival relative to 5.5 mM glucose controls (cell viability = 100%, mean ± SEM, *n* = 6). After Raw 264.7 cells were cultured for five days, TRAP-positive activity was measured at λ = 405 nm using a leukocyte acid phosphatase kit (E, mean ± SEM, *n* = 3), and TRAP-positive multinucleated osteoclasts were stained and visualized under light microscopy (**F**). Blue arrows indicate the differentiated osteoclasts. Magnification: 20-fold. Values in the bar graphs not sharing a common letter indicate significant difference at *p* < 0.05.

The cytotoxicity of coumarin was determined by using a MTT assay. Raw 264.7 cells seeded at a density of 1 × 10^4^ cells/mL on a 24-well were incubated in 5.5 mM glucose- or 33 mM glucose-loaded α-MEM and exposed to 1–20 μM coumarin for 48 h in the absence and presence of 50 ng/mL RANKL. Cells were treated with 1 mg/mL MTT solution and incubated at 37 °C for 3 h, resulting in the formation of an insoluble purple formazan product that was dissolved in 250 μL isopropanol. Optical density was measured using a microplate reader at λ = 570 nm. Coumarin at the doses of 1–20 μM did not cause apparent cytotoxicity (Figure 1B). The current experiments employed coumarin concentrations in the range of 1–20 μM.

### 2.3. Measurement of Tartrate-Resistant Acid Phosphatase (TRAP) Staining and Activity

Raw 264.7 macrophages were cultured for five days on chamber slides and incubated in an α-MEM containing 5.5 mM or 33 mM glucose with 50 ng/mL RANKL in the absence or presence of 1–20 μM coumarin. Cells were fixed with 4% formaldehyde and stained for 30 min with a commercially-available TRAP kit (Sigma-Aldrich Chemical). TRAP-positive multinucleated osteoclasts were visualized under light microscopy.

For the measurement of TRAP activity, cells were fixed with 4% formaldehyde for 10 min. Subsequently, the dried cells were incubated in 50 mM citrate buffer (50 mM citric acid and 50 mM sodium citrate (pH 4.5)) containing 5 mM 4-nitrophenylphosphate and 10 mM sodium tartrate for 1 h [27]. The reaction was terminated by adding 0.1 N NaOH. The absorption intensity was measured using a microplate reader at λ = 405 nm.

### 2.4. Western Blot Analysis

Western blot analysis was conducted using whole-cell lysates. Culture media and whole cell lysates were prepared in a lysis buffer containing 1 M β-glycerophosphate, 1% β-mercaptoethanol, 0.5 M NaF, 0.1 M Na_3_VO_4_, and protease inhibitor cocktail. Equal volume of culture media and cell lysates containing equal amounts of total proteins were electrophoresed on 8–15% sodium dodecyl sulfate-polyacrylamide gel electrophoresis and transferred onto a nitrocellulose membrane. Non-specific binding was blocked by soaking the membrane in a TBS-T buffer [50 mM Tris-HCl (pH 7.5), 150 mM NaCl and 0.1% Tween 20] supplemented for 3 h with 5% (*w*/*v*) skim milk or 3% bovine serum albumin. The membrane was incubated with a primary antibody to CAII, RANK, AGE, RAGE, V-ATPase, ClC7, cathepsin K, OPG, or collagen type I at 4 °C overnight. Following three washes with TBS-T buffer, the membrane was then incubated with a secondary antibody of goat anti-rabbit IgG, goat anti-mouse IgG, or donkey anti-goat IgG conjugated to HRP. Each protein level was determined by using enhanced chemiluminescent detection reagents (Millipore, Billerica, MA, USA) and Konica x-ray film (Konica Co., Tokyo, Japan). Incubation with anti-human β-actin was conducted for the comparative control.

### 2.5. Actin Ring Staining

Raw 264.7 cells on 24-well plates were incubated in an α-MEM containing 5.5 mM or 33 mM glucose with 50 ng/mL RANKL in the absence or presence of 1–20 μM coumarin for five days [27,28]. Cells were fixed in 4% formaldehyde for 10 min and washed with pre-warmed phosphate buffered saline (PBS). Subsequently, 10 units of the fluorescent dye rhodamine phalloidin was added to cells and incubated for 20 min. Nuclear staining was also conducted by using 1 mg/mL 4′,6-diamidino-2-phenylindole (DAPI). Fluorescent images were taken with an Axiomager optical fluorescence microscope.

### 2.6. Bone Resorption Assay

Bone resorption assay was performed by using a resorption assay kit (Cosmo Bio Co., Tokyo, Japan). Raw 264.7 cells were incubated in an α-MEM (5.5 mM or 33 mM glucose) with 50 ng/mL RANKL in the absence or presence of 1–20 μM coumarin. After culture for five days, the cells were washed in 5% NaOCl to remove the cells and the pit areas were measured [27]. The resorbed areas on the plate were visualized under light microscopy.

### 2.7. MC3T3-E1 Cell Culture and Osteoblast Differentiation

MC3T3-E1 cell line (ATCC CRL-2593) was obtained from the American Type Culture Collection, and cultured in an α-MEM supplemented with 10% FBS, 100 U/mL penicillin, and 100 μg/mL streptomycin at 37 °C with 5% CO_2_ in air. To differentiate MC3T3-E1 cells into osteoblasts [29,30], cells were seeded on 24-well plates at a density of 6.5 × 10^4^ cells, and were cultured in α-MEM containing 5.5 mM or 33 mM glucose supplemented with 10 mM β-glycerol phosphate, 50 μg/mL ascorbic acid, and 100 nM dexamethasone (differentiation media) for 21 days in the absence and presence of 1–20 μM coumarin. The culture media for cells were freshly replaced every three days.

For the measurement of MC3T3-E1 cytotoxicity of coumarin, cells were seeded in a 24-well plate at a density 6.5 × 10^4^ cells, and were treated with 1–20 μM coumarin for three days in 5.5 mM glucose- or 33 mM glucose-containing media. After cell culture with coumarin, 1 mg/mL MTT reagent was added to cells and incubated for 3 h at 37 °C with 5% CO_2_. Optical density was measured by using a microplate reader at λ = 570 nm.

### 2.8. Measurement of Alkaline Phosphatase (ALP) Activity and Staining

Our previous study showed that the ALP activity peaked between day 6 and day 9 during the 21-day differentiation process of MC3T3-E1 cells [30]. ALP activity was performed on day 7 of the MC3T3-E1 cell differentiation. Cells were lysed by 0.5% Triton X-100, followed by incubation with 0.5 M Tris–HCl (pH 9.9) containing 6 mM *p*-nitrophenyl phosphate and 1 mM MgCl_2_ at 37 °C for 2 h. The protein contents were determined, and the absorbance was read at λ = 405 nm in a microplate reader. ALP activity was expressed as nmol *p*-nitrophenyl phosphate (*p*NP) produced/min/mg protein.

The ALP staining was performed by using an ALP kit (Sigma-Aldrich Chemical). After 6-day culture protocols, cells were washed with PBS and fixed with 4% formaldehyde, rinsed with 0.05% Tris-buffered saline-Tween 20 (mixture of Tris-buffered saline and 0.1% Tween 20), stained under protection from direct light. The ALP staining was conducted by adding naphthol/Fast Red Violet solution for 30 min as a substrate for cells. Naphthol/Fast Red Violet solution is a mixture of Fast Red Violet (0.8 g/L) with a 4 mg/mL Naphthol AS-BI phosphate solution in 2 M AMPD buffer (pH 9.5). Images for the visualization of ALP and its staining intensity were measured using an optical Axiomager microscope system (Carl Zeiss, Oberkochen, Germany).

### 2.9. Alizarin Red S Staining

Alizarin Red S staining was employed in order to visualize bone nodule formation and calcium accumulation in osteoblasts. To measure calcium deposits [30], MC3T3-E1 cells were seeded in a 24-well plate at density 6.5 × 10^4^ cells in differentiation media containing 5.5 mM or 33 mM glucose for 21 days in the absence and presence of 1–20 μM coumarin. The culture medium was freshly changed every three days, and Alizarin Red S staining was done on the day 21. On the day 21, cells were rinsed in cold PBS, fixed with 70% ethanol at 4 °C for 1 h, and stained with 40 mM Alizarin red S dye (pH 4.2) for 10 min. The images for the visualization of calcium deposits and the staining intensity of Alizarin red S were measured using an optical Axiomager microscope system (Carl Zeiss).

### 2.10. Data Analysis

The results are presented as the mean ± standard error of the mean (SEM) for each treatment group. Statistical analyses were performed using Statistical Analysis Systems statistical software package (version 9.4, SAS Institute Inc., Cary, NC, USA). Significance was determined by one-way Analysis of Variance, followed by the Duncan range test for multiple comparisons. Differences were considered significant at *p* < 0.05.

## 3. Results

### 3.1. Promotion of Osteoclast Differentiation by Coumarin in High Glucose Cultures

The 2 day-incubation with 33 mM glucose increased the viability of RAW 264.7 macrophages, which was not influenced by non-toxic coumarin at doses of 1–20 μM (Figure 1C). In addition, treatment with 50 ng/mL RANKL alone enhanced the viability of Raw 264.7 macrophages in media containing 5.5 mM glucose, but not 33 mM glucose (Figure 1D).

This study found that glucose interfered with RANKL-mediated osteoclast differentiation, which was positively affected by coumarin. High glucose levels suppressed TRAP activity of RAW 264.7 macrophages exposed to RANKL, relative to 5.5 mM glucose (Figure 1E). In contrast, the TRAP activity was dose-dependently increased in 1–20 μM coumarin-treated macrophages exposed to RANKL. As expected, RANKL-induced macrophage differentiation into multinucleated osteoclasts was inhibited by elevated levels of glucose (Figure 1F). However, coumarin promoted the formation of TRAP-positive multinucleated cells.

**Figure 2:**
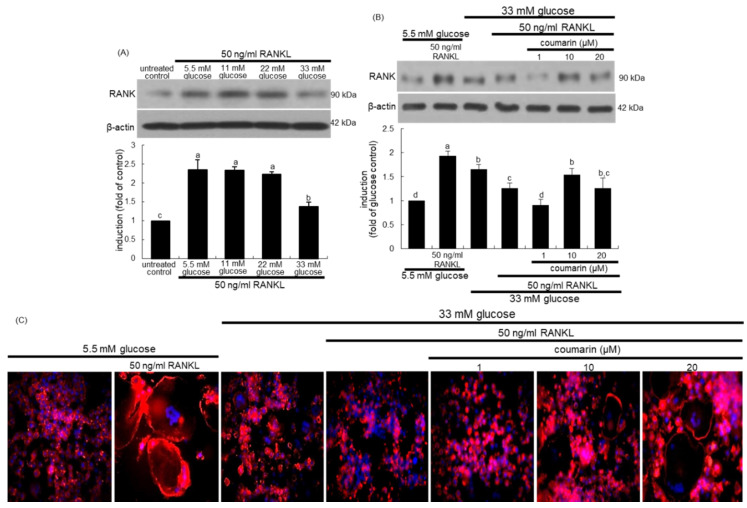
Receptor activator of NF-kappa B (RANK) induction by various concentrations of glucose (**A**), elevation of RANK induction (**B**), and osteoclast actin ring formation (**C**) by coumarin in glucose and RANKL-exposed Raw 264.7 cells. RAW 264.7 cells were cultured in α-MEM containing 5.5 mM glucose or 11-33 mM glucose in the absence and presence of 50 ng/mL RANKL or 1–20 μM coumarin for five days. The RANK induction was measured by western blot analysis using cell lysates with a primary RANK antibody (**A**,**B**). β-Actin protein was used as an internal control. Representative blots shown are typical of three independent experiments. The bar graphs (mean ± SEM) in the bottom panel represent quantitative results obtained from a densitometer. Values not sharing a letter are different at *p* < 0.05. RANKL-differentiated cells were fixed, and rhodamine phalloidin was added to fixed cells (**C**). Fluorescent images were taken with a fluorescence microscope. Original magnification of microscopic images (*n* = 3), 40-fold.

The interaction between the cell-surface receptor RANK and its ligand RANKL is essential for mature osteoclast formation [31]. Western blot data revealed that the induction of RANK in osteoclasts by RANKL was strongly inhibited by increasing the glucose concentrations in the media (Figure 2A). Treatment of RANKL-exposed Raw 264.7 macrophages with 10 μM coumarin in the presence of 33 mM glucose reversed the reduced RANK induction (Figure 2B).

### 3.2. Activation of High Glucose-Exposed Osteoclasts by Coumarin

This study found that coumarin ameliorated glucose-triggered osteoclastogenic dysfunction in RAW 264.7 macrophage-derived osteoclasts. The formation of actin rings was elevated in RANKL-treated macrophages, as evidenced by phalloidin staining (Figure 2C). However, high glucose failed to form an actin ring structure in RANKL-induced Raw 264.7 macrophages (Figure 2). In contrast, exposure to ≥10 μM coumarin restored the formation of actin rings. Thus, coumarin may boost the formation of sealing zone-like structures for optimal bone resorption in diabetic osteoclasts.

**Figure 3:**
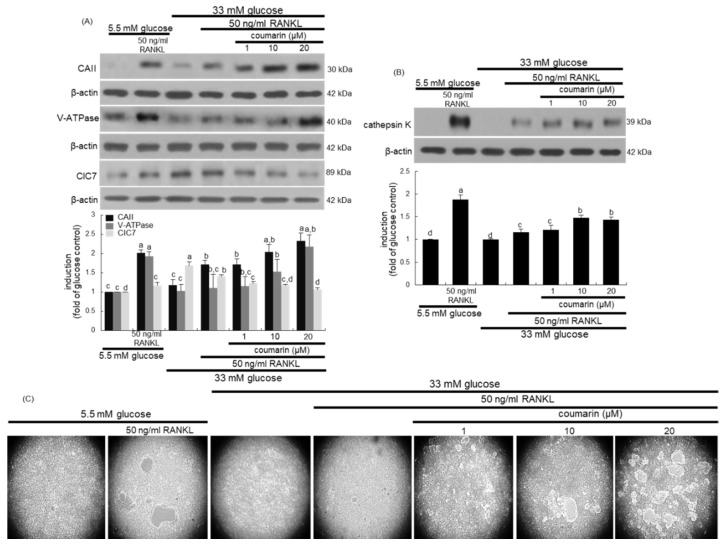
Western blot data showing the effects of coumarin on the induction of carbonic anhydrase II (CAII), vacuolar H^+^-ATPase (V-ATPase), chloride channel 7 (ClC7), and cathepsin K in glucose-exposed osteoclasts. Raw 264.7 cells were cultured in α-MEM containing 5.5 mM glucose or 33 mM glucose with 50 ng/mL RANKL in the absence and presence of 1–20 μM coumarin for five days. Cell lysates were subject to western blot analysis with a primary antibody against CAII, V-ATPase, ClC7, and cathepsin K (**A**,**B**). Representative blot data were obtained from three independent experiments, and β-actin protein was used as an internal control. The bar graphs (mean ± SEM) in the bottom panel represent quantitative results obtained from a densitometer. Values not sharing a letter are different at *p* < 0.05. The osteoclast bone resorption was assayed by using a commercially available bone resorption assay kit (**C**). The resorbed areas on the plate were visualized under light microscopy with 20-fold magnification.

This study further examined whether coumarin affected bone resorption normally in diabetic osteoclasts by maintaining optimal acidification of resorptive lacunae. The activities of osteoclastic CAII and V-ATPase in proton generation were strongly elevated in RANKL-treated osteoclasts (Figure 3A). Such expression was notably suppressed in diabetic osteoclasts, while the treatment with 1–20 μM coumarin dose-dependently enhanced the induction of CAII and V-ATPase (Figure 3A). The intracellular chloride channels of the ClC7 gene family located at the ruffled border membrane of osteoclasts are to maintain electrostatic neutralization during osteoclast acid secretion [32]. In contrast to the induction of CAII and V-ATPase, the CIC7 induction was increased in glucose-loaded osteoclasts, which was inhibited by coumarin in the presence of RANKL (Figure 3A). Furthermore, the lysosomal protease cathepsin K induction was highly enhanced by the RANKL presence, which was markedly abrogated in high glucose-loaded macrophages (Figure 3B). However, the presence of ≥10 μM coumarin prompted cathepsin K induction in diabetic osteoclasts.

This study investigated the effects of coumarin on real-time bone-resorbing activity of RANKL- and glucose-exposed macrophages incubated on calcium phosphate-coated plates. The mature normal osteoclasts created several resorption pits, whereas glucose-loaded osteoclasts reduced the pit areas (Figure 3C). The bone absorption was greatly enhanced by the addition of 1–20 μM coumarin to glucose-loaded osteoclasts.

### 3.3. Disruption of AGE–RAGE Interaction by Coumarin

**Figure 4:**
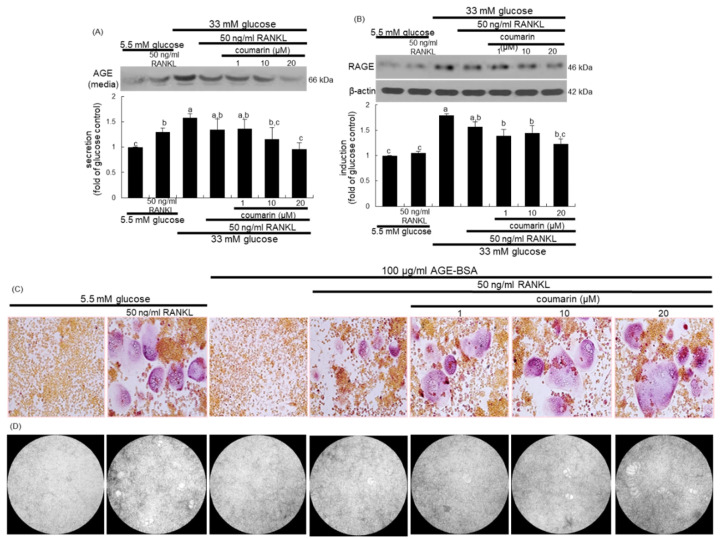
Inhibition of glucose-induced advanced glycation end products (AGE) secretion (**A**) and receptor for AGE (RAGE) expression (**B**), and elevation of AGE-induced osteoclast differentiation (**C**), and bone resorption (**D**) by coumarin in Raw 264.7 cells. Cells were cultured in α-MEM media containing 5.5 mM glucose, 33 mM glucose, or 100 μg/mL AGE with 50 ng/mL RANKL in the absence and presence of 1–20 μM coumarin for five days. Cell media and lysates were subject to western blot analysis with a primary antibody against AGE and RAGE. Representative blot data were obtained from three independent experiments, and β-actin protein was used as an internal control. The bar graphs (mean ± SEM) in the bottom panel represent quantitative results obtained from a densitometer. Values not sharing a letter are different at *p* < 0.05. After cells were cultured with 100 μg/mL AGE–bovine serum albumin (BSA) for five days, cells were fixed and stained using a leukocyte acid phosphatase kit (**C**). TRAP-positive multinucleated osteoclasts were visualized under light microscopy. The osteoclast bone resorption was assayed by using a commercially available bone resorption assay kit (**D**). The resorbed areas on the plate were visualized under light microscopy. Magnification: 20-fold.

The glucose loading increased the AGE secretion and RAGE induction in macrophages, which was sustained during osteoclast differentiation by RANKL (Figure 4A,B). However, the treatment with 1–20 μM coumarin dose-dependently attenuated the AGE secretion and RAGE induction in RANKL-activated Raw 264.7 macrophages exposed to glucose. The AGE–RAGE interaction disrupts bone mineral resorption at the cell fusion stage of osteoclast [33].

This study investigated the role of AGE in osteoclast differentiation in the fusion stage. As expected, the TRAP staining revealed that RANKL strongly induced TRAP-positive multinucleated osteoclasts (Figure 4C). In contrast, treatment with 100 μg/mL AGE–BSA abrogated the induction of TRAP-positive multinucleated osteoclasts, indicating that AGE inhibited osteoclast formation at the fusion stage. When coumarin was added to AGE–BSA-treated macrophages, RANKL-mediated induction of multinucleated osteoclasts was markedly restored (Figure 4C). Thus, coumarin may trigger osteoclast differentiation suppressed by AGE generated from diabetic macrophages. In addition, exposure to 100 μg/mL AGE–BSA failed to resorb fluoresceinated calcium phosphate in the presence of RANKL (Figure 4D). However, the supplementation of 20 μM coumarin increased the bone-resorbing activity of AGE–BSA-exposed osteoclasts.

### 3.4. Enhancement of Bone Mineralization by Coumarin

**Figure 5:**
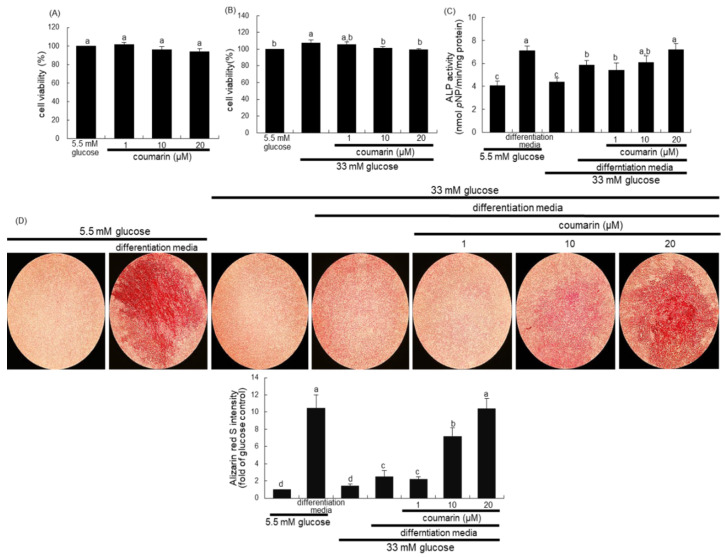
Osteoblastic MC3T3-E1 cell toxicity of coumarin (**A**,**B**), and elevation of alkaline phosphatase (ALP) activity (**C**) and calcium deposit (**D**) in osteoblastic MC3T3-E1 cells by coumarin. MC3T3-E1 cells were cultured for three days with and without 1–20 μM coumarin. Cell viability was measured by the MTT assay and expressed as percent cell survival relative to untreated glucose controls (cell viability = 100%, mean ± SEM, *n* = 3). The ALP enzyme activity in media was expressed as nmol *p*-nitrophenyl phosphate (*p*NP)/min/mg protein (**C**). Absorbance was measured at λ = 405 nm and compared with *p*-nitrophenol standard (mean ± SEM, *n* = 7). MC3T3-E1 cells were cultured in differentiation media containing 5.5 mM glucose or 33 mM glucose in the presence of 1–20 μM coumarin for six days (**C**). Extracellular matrix (ECM) calcium deposit (bone nodule formation) was measured by Alizarin Red S staining. MC3T3-E1 osteoblasts were cultured in differentiation media containing 5.5 mM glucose or 33 mM glucose in the presence of 1–20 μM coumarin for 21 days. Microphotographs were representative of 21 day-grown osteoblasts on the wells. Heavy reddish staining of Alizarin Red S is proportional to the area of mineralized ECM. Its intensity was also measured. The calcium deposits were visualized under light microscopy, and the ALP staining intensity was measured using an optical Axiomager microscope system. Magnification: 10-fold. Values in bar graphs not sharing a letter indicate significant difference at *p* < 0.05.

There was no alteration in the viability of MC3T3-E1 cells following exposure to 1–20 μM coumarin (Figure 5A). Non-toxic coumarin corrected the increased MC3T3-E1 cell proliferation by 33 mM glucose (Figure 5B). Alizarin Red S staining was employed in order to visualize bone nodule formation and calcium accumulation in osteoblasts. ALP is crucial for bone mineralization as a useful early- and mid-stage biochemical marker of bone formation [34]. The ALP activity, expressed as *p*NP production/min/mg cell protein, was markedly enhanced in MC3T3-E1 cells cultured in osteogenic differentiation media (Figure 5C). The colorimetric enzyme assay revealed that high glucose suppressed the ALP activity of MC3T3-E1 cells (Figure 5C). However, treatment with 1–20 μM coumarin induced the ALP activity, which was reduced in diabetic MC3T3-E1 cells.

Alizarin Red S staining revealed no calcium deposit in undifferentiated MC3T3-E1 cells, whereas strong staining of Alizarin Red S was detected in cells cultured in differentiation media for 21 days (Figure 5D). In contrast, calcium accumulation and mineralized bone nodule formation were highly reduced in diabetic MC3T3-E1 cells cultured even in osteogenic differentiation media (Figure 5D). When ≥10 μM coumarin was administered to diabetic MC3T3-E1 cells in osteogenic media, calcium deposits were significantly enhanced. Taken together, coumarin may boost differentiation and bone mineralization of diabetic osteoblasts.

**Figure 6:**
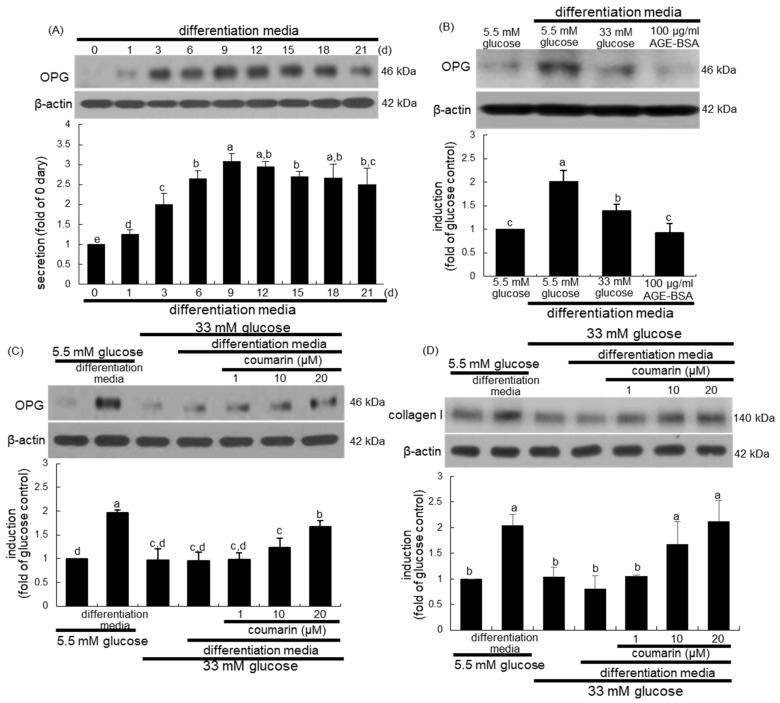
Temporal induction of osteoprotegerin (OPG, **A**), and elevation of induction of OPG (**B**,**C**) and collagen type I (**D**) by coumarin in osteoblastic MC3T3-E1 cells. MC3T3-E1 cells were cultured in differentiation media containing 5.5 mM glucose, 33 mM glucose, or 100 μg/mL AGE–BSA in the presence of 1–20 μM coumarin for up to 21 days. Cell lysates were subject to western blot analysis with a primary antibody against OPG and collagen I. Representative blot data were obtained from three independent experiments, and β-actin protein was used as an internal control. The bar graphs (mean ± SEM) in the bottom panel represent quantitative results obtained from a densitometer. Values in bar graphs not sharing a letter indicate significant difference at *p* < 0.05.

Osteogenic OPG released by osteoblasts inhibits osteoclast formation and function by binding to RANKL and preventing it from binding to RANK [35]. When MC3T3-E1 cells were cultured in differentiation media for up to 21 days, the cellular expression of OPG temporally increased and peaked on days 9 to 12, and tended to decline thereafter until day 21 (Figure 6A). Furthermore, treatment with 100 μg/mL AGE–BSA attenuated the OPG induction in MC3T3-E1 cells cultured in osteoblastogenic differentiation media (Figure 6B). When MC3T3-E1 cells were exposed to 33 mM glucose in differentiation media for nine days, the OPG induction was markedly suppressed (Figure 6C). Western blot analysis showed that the treatment of MC3T3-E1 cells with 1–20 μM coumarin for nine days in 33 mM glucose-loaded osteogenic media dose-dependently stimulated OPG induction (Figure 6C). In addition, the induction of collagen type I was promoted in MC3T3-E1 cells cultured in differentiation media, while glucose inhibited collagen I induction during the nine day-osteogenic differentiation process (Figure 6D). However, exposure to 1–20 μM coumarin boosted collagen induction in diabetic osteoblasts (Figure 6D).

### 3.5. Elevation of Bone-Forming Activity in AGE-Exposed Osteoblasts by Coumarin

**Figure 7:**
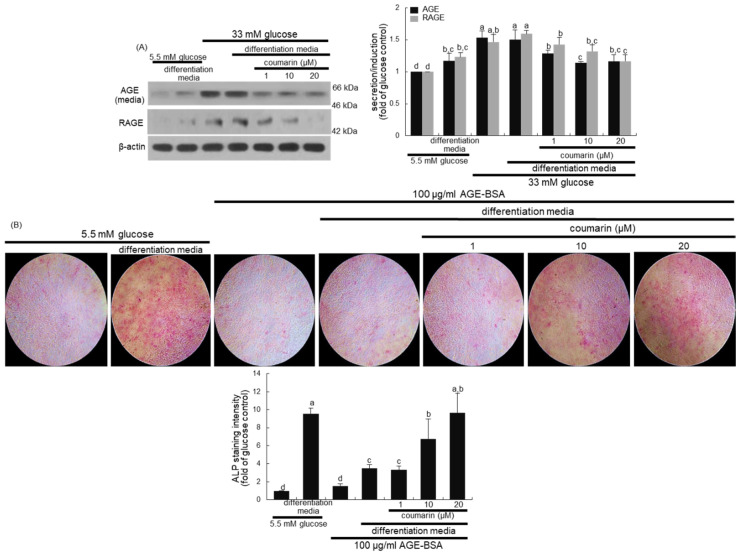
Inhibition of advanced glycation end-product (AGE) secretion and receptor for AGE (RAGE) induction (**A**) in osteoblastic MC3T3-E1 cells by coumarin. MC3T3-E1 cells were cultured for nine days in the presence of 1–20 μM of coumarin. Cell media and lysates were subject to western blot analysis with a primary antibody against AGE and RAGE. Representative blot data were obtained from three independent experiments, and β-actin protein was used as an internal control. The bar graphs (mean ± SEM) in the bottom panel represent quantitative results obtained from a densitometer. MC3T3-E1 cells were cultured in differentiation media containing 5.5 mM glucose or 100 μg/mL AGE–bovine serum albumin (BSA) in the presence of 1–20 μM coumarin on glass chamber plates for six days (**B**). Alkaline phosphatase (ALP) staining was proportional to the content of ALP enzyme activity from MC3T3-E1 cells. The intensity of ALP staining (mean ± SEM, *n* = 4) was measured using an optical Axiomager microscope system (**B**). The ALP staining was visualized under light microscopy. Magnification: 10-fold. Values not sharing a letter are different at *p* < 0.05.

High glucose greatly elevated AGE secretion and RAGE induction in MC3T3-E1 cells, which was sustained even during osteogenic differentiation (Figure 7A). When diabetic MC3T3-E1 cells in differentiation media were treated with ≥10 μM coumarin, the production of AGE and RAGE was significantly abrogated. Long-term treatment of AGE inhibits osteogenic function and enhances osteoclastogenesis via interaction with RAGE [36].

This study further found that the interaction between AGE and RAGE disrupted osteoblastic differentiation and bone formation. A weak staining for the basal ALP activity was detected in undifferentiated MC3T3-E1 cells, as evidenced by Fast Red Violet staining (Figure 7B). In contrast, heavy lilac staining was observed in differentiated cells, indicative of increased ALP secretion into the ECM (Figure 7B). However, exposure to 100 μg/mL AGE–BSA abolished ALP staining in cells in the differentiation media (Figure 7B). Addition of 1–20 μM coumarin to cells exposed to AGE–BSA resulted in enhanced staining of ALP in a dose-dependent manner. 

## 4. Discussion

To maintain normal skeletal balance, the aged or damaged bone is adequately replaced by an equivalent amount of new bone [37]. Therefore, the two processes of bone resorption and formation are tightly coupled via the RANK/RANKL/OPG system during the bone remodeling. Aberrant bone remodeling results in bone diseases such as osteoporosis [8,9]. Several metabolic diseases accompany pathological features in the bone due to impaired bone remodeling [8,10]. Bone turnover is reduced or disrupted in diabetes mellitus, in parallel with increased risk of bone fractures and reduced mineralization [12,16]. One study showed that high glucose inhibits RANKL-induced osteoclastogenesis in diabetes by inhibiting redox-sensitive NF-κB activity [38]. The current study revealed that high glucose consistently reduced the osteoclast differentiation and bone resorption, and also suppressed the differentiation of bone-forming osteoblasts, leading to the disruption of bone turnover. These results suggested the role of glucose in diabetes-associated bone diseases. Similarly, diabetes leads to reduced bone turnover, resulting in increased rate of bone loss, altered bone structure, and predisposition to bone fragility [11].

Anti-osteoporotic medications such as alendronate and raloxifene appear to be equally effective in diabetic and non-diabetic individuals with reduced bone turnover [21]. The anti-diabetic thiazolidinediones inhibit osteoclastic bone resorption regardless of their adipogenic effects [39]. Conventional therapies for the treatment and prevention of osteoporosis are associated with significant drawbacks and are limited by adverse effects such as burning sensation and gastrointestinal tract disturbances. Thus, the use of natural compounds for multifactorial dysmetabolic bone diseases is an effective alternative to overcome side effects of conventional therapies. This study investigated whether coumarin ameliorated bone turnover and remodeling under diabetic conditions. Coumarins derived from fruits of *Cnidium monnieri* exhibited beneficial anti-osteoporotic activity by inhibiting osteoclast activation [25]. In addition, coumarin derivatives of bergapten and methoxsalen prevent diabetic osteoporosis by suppressing osteoclastogenic gene expression and bone resorption in diabetic bone tissues [40]. However, little is known about the effects of coumarin on diabetes-associated abnormal bone turnover mediated via activation and differentiation of osteoclasts and osteoblasts. In the current study, coumarin enhanced osteoclastogenic activation of macrophages derailed by loading glucose. Additionally, this compound accelerated RANK induction and actin ring formation, accompanying cathepsin K-mediated bone resorption of diabetic osteoclasts. Accordingly, coumarin may ameliorate diabetic dysfunction of osteoclastogenesis concomitantly with optimal enzymatic activity at the low pH during the acidification of resorption lacuna. A recent study reported that psoralen accelerates bone fracture healing through activation of both osteoclasts and osteoblasts via preferential signaling of ERK, but not JNK or p38 MAPK [41].

Hyperglycemia in diabetes directly promotes fat accumulation in the marrow cavity, leading to increased fracture risk [42]. Several plant polyphenols such as isoflavones and quercetin have been extensively used as a source of therapeutic agents enhancing bone anabolic effects [43,44]. Several in vitro studies and in vivo animal models establish the osteoblastogenic significance of plant polyphenolic compounds exhibiting antioxidant properties and anti-inflammatory actions. Nevertheless, adequate evidence in the context of bone health in diabetes is unavailable. A study showed that coumarin derivatives of imperatorin and bergapten enhance ALP activity, collagen type I synthesis, bone morphogenetic protein-2 expression, and bone nodule formation in primary cultured osteoblasts and tibia tissues [26]. However, no studies have consistently correlated the effects of coumarin with bone turnover in diabetes. This study showed that coumarin promoted differentiation and calcium deposit of osteoblastic MC3T3-E1 cells in parallel with elevated ALP activity and collagen I expression suppressed by glucose loading. Collectively, coumarin may enhance the bone quality and stimulate bone turnover under glucose toxicity.

Increasing evidence suggests that AGE–RAGE interaction may induce functional and structural impairment of bone typical of osteoporosis [45,46]. The aberrant changes in cellular redox state and oxidative stress result in disruption of normal bone remodeling, leading to pathological bone loss [47]. The ligand–RAGE interaction leads to the generation of reactive oxygen species and consequent downstream signal transduction and regulation of gene expression [48]. In addition, AGE may exhibit adverse pro-inflammatory activities in osteoporosis [46]. The current study suggests that the AGE induction in diabetic bone cells contributes to the uncoupling of osteoclast-mediated bone resorption and osteoblast-mediated bone formation, thereby disrupting normal remodeling. Thus, it can be speculated that coumarin ameliorated bone turnover by hampering the AGE–RAGE interaction via anti-oxidative mechanism and anti-inflammatory activity. Inflammation impairs normal bone remodeling, in which inflammatory cytokines are central to the pathogenesis of bone loss [49]. The AGE–RAGE signaling pathway plays a role in diabetic complications including diabetic osteopathy [45]. Furthermore, in this study, coumarin directly interfered with the tight coupling of bone resorption and formation by suppressing bone turnover-related markers of RANK and OPG. Weakening of bone tissues is related to AGE accumulation in the bone collagen fibers, leading to impaired bone cell function and ECM complications [45,46].

## 5. Conclusions

The current study demonstrated that coumarin enhanced multinucleated osteoclast formation and bone resorption by diabetic osteoclasts through normalizing RANK-RANKL signaling. Concurrently, coumarin promoted osteoblastogenic differentiation in parallel with calcium deposit, ALP activity, and collagen type I induction in MC3T3-E1 cells derailed by glucose- and AGE-loading. Both the processes of bone resorption and formation appeared to be tightly coupled via the RANKL/OPG system stabilized by coumarin. Furthermore, the hyperglycemia-induced AGE–RAGE signaling pathway disrupted bone turnover under glucose toxicity, which is one of the molecular mechanisms of action of coumarin underlying the amelioration of bone turnover and remodeling. Few standard therapies are available for the treatment and prevention of diabetic osteopathy. Accordingly, a possible protective effect by coumarin in this multifactorial dysmetabolic disease represents a potential alternative to conventional therapies due to diminished side effects. However, studies with consistent findings related to coumarin consumption by humans have yet to be reported. 

## Abbreviations

AGE	advanced glycation end-product
AGE–BSA	advanced glycation end product–bovine serum albumin
ALP	alkaline phosphatase
CAII	carbonic anhydrase II
ClC7	chloride channel 7
ECM	extracellular matrix
OPG	osteoprotegerin
RAGE	receptor for advanced glycation end-product
RANK	receptor activator of nuclear factor-kappa Β
RANKL	receptor activator of nuclear factor-kappa Β ligand
TRAP	tartrate-resistant acid phosphatase
V-ATPase	vacuolar H^+^-ATPase
